# Active ageing in the digital era: digital literacy, social networks, and mental health among older adults in China

**DOI:** 10.3389/fpsyg.2026.1767846

**Published:** 2026-03-25

**Authors:** Ling Zong, Kexin Zhou

**Affiliations:** 1 School of Health, Jiangsu Vocational Institute of Commerce, Nanjing, China; 2 National Research Center for Resettlement, Hohai University, Nanjing, China

**Keywords:** digital literacy, mental health, older adults, psychological wellbeing, social networks, sustainable development goals

## Abstract

**Introduction:**

In the context of rapid population ageing and the global pursuit of Sustainable Development Goal 3 (Good Health and Wellbeing), understanding how digital capabilities shape psychological wellbeing among older adults has become increasingly important. This study aims to examine the impact of digital literacy on the mental health of middle-aged and older adults in China and to explore the mediating role of social networks in this relationship.

**Methods:**

This study uses data from 9,507 respondents drawn from the fourth wave of the China Longitudinal Ageing Study (CLASS) conducted in 2020. Ordinary least squares (OLS) regression and generalized method of moments (GMM) estimation are employed to examine the association between digital literacy and psychological wellbeing. A mediation model is further constructed to test whether social networks serve as an underlying mechanism.

**Results:**

The results indicate that the average mental health score of older adults is 20.09, while the mean digital literacy score is 0.203. Digital literacy has a significant positive effect on psychological wellbeing (OLS: *β* = 0.951, *p* < 0.01; GMM: *β* = 0.607, *p* < 0.01), and social networks partially mediate this relationship, with an indirect effect of 0.035, accounting for approximately 3.8% of the total effect. In addition, advanced age (*β* = −3.971, *p* < 0.01), male gender (*β* = −0.193, *p* < 0.01), non-agricultural household registration (*β* = −0.332, *p* < 0.01), and dependence on others for daily activities (*β* = −1.107, *p* < 0.01) are negatively associated with mental health. By contrast, higher educational attainment (*β* = 0.042, *p* < 0.01), greater income (*β* = 0.003, *p* < 0.01), favorable marital status (*β* = 0.291, *p* < 0.01), and having more children (*β* = 0.052, *p* < 0.01) are positively related to psychological wellbeing.

**Conclusion:**

The findings suggest that digital literacy enhances psychological wellbeing among middle-aged and older adults both directly and indirectly by strengthening social connectedness. This study highlights the importance of integrating digital inclusion strategies into mental health promotion systems for ageing populations and emphasizes the role of supportive social networks in promoting active ageing and sustainable wellbeing in the digital era.

## 1 Introduction

Population ageing has become a pervasive and irreversible global trend. Many European countries and regions worldwide are projected to experience substantial increases in the proportion of older adults in the coming decades. For instance, by 2,100, more than 30% of the population in the European Union is expected to be aged 65 years or above. Currently, Europe is home to approximately 176 million older persons, reflecting the accelerating pace of demographic ageing worldwide (Araújo et al., 2025). China is undergoing a similar transformation: by 2024, the number of people aged 60 and above reached 310 million, representing 22% of the total population (Du and Tan, 2025). In China, rapid socioeconomic development, shrinking family sizes, and rising numbers of empty-nest households have reshaped traditional support structures, generating new challenges for maintaining the psychological wellbeing of older adults.

Concurrently, digital technologies have penetrated deeply into daily life, including among older populations. According to the National Bureau of Statistics, the number of internet users aged 60 and above in China reached 156 million in 2024 (Zhou and Xia, 2025). However, older adults have not benefited equally from digital transformation. Due to later exposure to digital technology and generally lower digital literacy, many older adults face heightened vulnerability in navigating digital platforms (Zhang and Tao, 2025). This vulnerability contributes not only to a deficit in digital competencies but also to forms of digital exclusion that limit access to essential public services and interpersonal communication, creating a “dual alienation” that undermines dignity and autonomy in later life (Zhu, 2023). Empirical studies further indicate that such structural exclusion generates direct and adverse consequences for mental health. Even when older adults attempt to participate in the digital environment, the outcomes can be ambivalent—enhanced social engagement may coexist with increased psychological stress.

Against this backdrop, promoting the health, wellbeing, and social inclusion of older adults has become a central component of global ageing governance and an essential pathway for achieving the United Nations Sustainable Development Goal 3 (SDG 3): ensuring healthy lives and promoting wellbeing for all at all ages (Li and Shao, 2025). Mental health, in particular, is widely recognized as a multidimensional construct. Foundational psychological theories—such as Allport’s mature personality, Maslow’s self-actualizing person, Rogers’s fully functioning person, Fromm’s productive person, and Frankl’s self-transcendent person—underscore that mental health encompasses not only emotional states but also personal growth, meaningful engagement, and relational functioning (Chen and Yao, 2005). The World Health Organization further defines mental health as a state of wellbeing in which individuals realize their abilities, cope with normal stresses, work productively, and contribute to their communities (Laidlaw et al., 2025). Existing research identifies multiple determinants of mental health among older adults, including demographic characteristics, socioeconomic status, social support networks, and physical health conditions (Sun, 2023; Wang and Li, 2024; Du et al., 2023; Li, 2025).

Digital literacy refers to the comprehensive set of competencies that enable individuals to effectively integrate digital technologies into their daily lives and learning processes in the digital era. It encompasses multiple dimensions, including information acquisition, content creation, technological application, interactive communication, and innovative practice, and serves as a foundational capability for individuals to participate in digital society and share the benefits of digital development. With the increasing prominence of the “digital divide,” a growing body of research has begun to examine the impact of digital technology access and use on the psychological wellbeing of older adults (Chong and Chiu, 2023; Feng et al., 2025; Liu and Li, 2024). The existing literature has gradually developed two representative theoretical perspectives. The social augmentation effect suggests that internet use enhances life satisfaction and psychological wellbeing by facilitating social connections, access to information, and recreational activities (Wang et al., 2025). Conversely, the displacement effect posits that excessive or maladaptive use of digital technology may reduce offline interactions, aggravate social isolation, and negatively influence mental health (Li et al., 2025). However, most studies have focused on internet usage as a binary behavioral indicator, overlooking the broader concept of digital literacy, which encompasses skills, cognitive capabilities, and the ability to apply digital tools effectively. Furthermore, limited research has examined the mechanisms through which digital literacy influences mental health, particularly in terms of social network pathways.

Accordingly, this study pursues three objectives. First, building upon established theoretical frameworks and incorporating the specific characteristics of older adults in China, we construct a multidimensional digital literacy evaluation system and apply the entropy method for objective weighting, thereby enhancing the scientific rigor and measurement validity of the index. Second, we develop a mediation model to systematically examine the underlying mechanisms through which digital literacy influences older adults’ mental health. Third, using nationally representative microdata from the 2020 China Longitudinal Aging Social Survey (CLASS), we conduct empirical analyses to provide robust evidence from the Chinese context to inform relevant theoretical discussions.

By addressing these questions, the study advances theoretical understanding of how digital competencies shape psychological wellbeing in later life, while also providing empirical evidence to support the design of targeted policies that promote digital inclusion and strengthen social support systems. These findings contribute to the broader discourse on active ageing and offer practical insights for advancing SDG 3 by enhancing the wellbeing and social participation of older adults in the digital era.

## 2 Theoretical analysis and research hypotheses

### 2.1 The impact of digital literacy on mental health in older adults

A synthesis of existing research suggests that digital literacy is not merely a set of technical operational skills, but rather a multidimensional, composite, and foundational competency framework. It encompasses not only the ability to access and operate digital tools, but also capacities for information filtering and evaluation, content creation, problem-solving, critical thinking, and communication and collaboration in digital environments. At the functional level, digital literacy has been characterized as a “survival skill in the digital era” and as a key asset in the information society.

However, for older adults, the meaning of digital literacy carries distinct implications. It extends beyond technical proficiency to include the capacity to meet age-specific needs, such as maintaining social connectedness, accessing health information, and coping with loneliness. Digital literacy becomes a meaningful resource for mental wellbeing only when it enables older adults to engage in video communication with their children, obtain medical and health information, and participate in online community activities. In this sense, digital literacy functions not simply as a technological skill set, but as a socially embedded capability linked to psychological resilience and social integration.

Regarding the impact of digital technology on older adults’ mental health, two classical theoretical perspectives have emerged. The social augmentation effect posits that digital technologies expand social networks and increase opportunities for social participation, thereby enhancing mental health. In contrast, the displacement effect argues that digital engagement may crowd out face-to-face interactions, weaken authentic emotional bonds, and consequently undermine psychological wellbeing. Nevertheless, these theoretical perspectives were largely developed within Western individualistic cultural contexts, and their applicability to Chinese older adults requires careful reconsideration.

Within the context of China’s familism-oriented cultural tradition, digital technology use is often centered on maintaining family relationships. This orientation may amplify the social augmentation effect—for example, through intergenerational communication in family chat groups—but may also intensify the displacement effect if online communication substitutes for in-person visits. Moreover, China’s pronounced urban–rural digital divide exposes rural older adults to a higher risk of digital exclusion, suggesting that improvements in digital literacy may generate comparatively stronger marginal augmentation effects in these settings. Accordingly, this study argues that the impact of digital literacy on older adults’ mental health is not unidirectional, but contingent upon broader cultural and structural conditions.

From a mechanistic perspective, digital literacy may influence mental health through both psychological and social pathways. Psychologically, the ability to use digital tools can enhance self-efficacy and perceived control—critical psychological resources for stress reduction and emotional wellbeing. Socially, digital literacy helps older adults overcome structural barriers associated with the digital divide, facilitating sustained social participation and access to information, both of which are recognized as key social determinants of health (Chen and Wang, 2025). Therefore, digital literacy is hypothesized to be a significant predictor of improved mental health among older adults (Yan et al., 2025). Based on the above theoretical analysis, this study proposes the following hypothesis:

*H1*: Higher levels of digital literacy are associated with better mental health among older adults.

### 2.2 Mediating role of social networks

Social support networks in this study refer to the functional dimension of social networks, specifically the supportive interactions embedded within interpersonal ties that provide emotional, instrumental, and informational resources (Chen and Wang, 2025). While social networks generally denote the structural configuration of social relationships, social support captures the functional resources exchanged within these ties. Given data availability, this study operationalizes social networks through friend-based social support, which serves as a proxy for the supportive function of older adults’ social networks.

**Figure 1:**
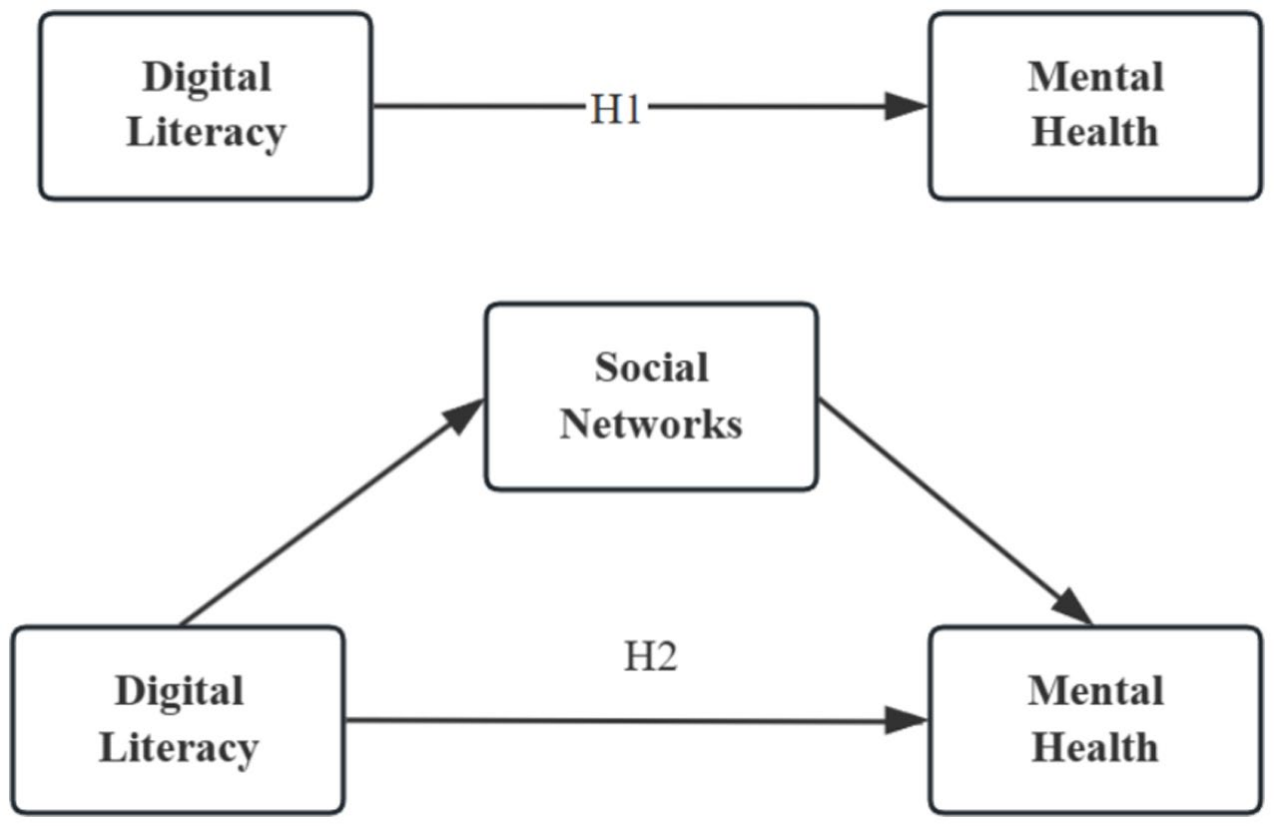
Conceptual framework illustrating the hypothesized direct and indirect effects of digital literacy on older adults’ mental health. The model presents the proposed mediation mechanism through social networks and serves as a theoretical framework guiding the empirical analysis. Arrows indicate hypothesized directional relationships.

Existing research suggests that the positive effect of digital literacy on mental health may not operate solely through a direct pathway; rather, part of its influence may be mediated by the strengthening of social support networks. From a theoretical perspective, social convoy theory posits that individuals are surrounded by dynamic layers of social relationships across the life course that provide continuous support resources. Digital literacy may enhance older adults’ ability to maintain existing ties and establish new connections by expanding communication channels and interaction modes, thereby reinforcing their supportive social convoy. Socioemotional selectivity theory further argues that as individuals age, they prioritize emotionally meaningful relationships. Proficiency in digital technologies enables older adults to sustain emotionally significant bonds with family members and close contacts, strengthening the supportive quality of their social networks and promoting emotional wellbeing. Moreover, according to the stress-buffering model of social support, supportive relationships mitigate the adverse psychological consequences of stress. By enhancing supportive exchanges within social networks, digital literacy may indirectly improve mental health outcomes among older adults (see Figure 1).

This mechanism is particularly salient in the Chinese context. Chinese society is characterized by strong familism, where intergenerational support constitutes a central component of older adults’ support networks. Digital literacy facilitates parent–child interaction and emotional exchange through platforms such as WeChat, thereby reinforcing family-based supportive ties. Meanwhile, pronounced urban–rural disparities in digital infrastructure mean that limited digital competence may exacerbate social exclusion. Strengthening supportive social connections through digital engagement may therefore reduce loneliness and enhance social integration, ultimately contributing to better mental health (Qu, 2016). Accordingly, this study proposes the following hypothesis:

*H2*: Digital literacy positively affects older adults’ mental health through the strengthening of social networks.

## 3 Research design

### 3.1 Data and variable

This study utilizes data from the fourth wave of the China Longitudinal Aging Social Survey (CLASS), conducted in 2020 by the Institute of Gerontology at Renmin University of China. CLASS is a nationally representative, longitudinal survey project that initiated its first national baseline in 2014, with follow-up waves administered in 2016, 2018, and 2020. The survey targets individuals aged 60 years and above within household units, with the final sample covering 28 provinces across China. The 2020 dataset represents the most recent follow-up wave, comprising an initial sample of 11,398 respondents. After excluding observations with missing values in key variables, the analytical sample consists of 9,507 individuals.

### 3.2 Variable definitions

#### 3.2.1 Dependent variable

The dependent variable in this study is older adults’ mental health status, encompassing both positive and negative psychological states. Mental health is measured using the revised nine-item Center for Epidemiologic Studies Depression Scale (CES-D) developed by Silverstein et al., which has been widely validated for research on older populations. The scale consists of nine items assessing respondents’ emotional condition, life attitudes, appetite, and sleep quality. Each item is rated on a three-point scale (1 = rarely, 2 = sometimes, 3 = often). After reverse coding the negatively worded items, the item scores are summed to generate a total mental health score ranging from 9 to 27. Higher scores indicate fewer depressive symptoms and thus better mental health. In this study, the Cronbach’s *α* coefficient of the scale is 0.695, suggesting acceptable internal consistency reliability (Jiang and Luo, 2024; Park and Lee, 2021). It should be noted that this study adopts the standard CES-D scoring procedure (i.e., equal-weight summation) to ensure comparability with international research and to avoid potential validity loss resulting from reweighting the scale items.

#### 3.2.2 Core independent variable: digital literacy

Numerous studies measure digital literacy using a single indicator, such as “whether the internet is used,” which may provide a limited perspective (Zhang et al., 2025). To comprehensively assess the digital literacy level of older adults and mitigate potential bias from unidimensional measurement, this study developed a multidimensional digital literacy index. Specifically, digital literacy was categorized into four dimensions: digital infrastructure, operational competence, communicative competence, and informational competence (Zhang et al., 2024), as detailed in Table 1. Since digital literacy is conceptualized as a composite capability constructed from multiple observable indicators rather than a latent psychological construct, this study employs the entropy weighting method to objectively determine the weights of each indicator. The entropy method assigns weights according to the degree of variation and information content of each indicator, thereby minimizing the subjective bias inherent in equal weighting approaches and avoiding the multicollinearity issues often associated with principal component analysis. Prior to index construction, all indicators are normalized using the min–max method to eliminate the effects of inconsistent measurement scales. The specific calculation procedure is as follows: first, the original indicators are standardized through min–max normalization; second, the information entropy of each indicator is calculated to derive its corresponding weight; finally, a weighted aggregation is performed to obtain the composite digital literacy index. The index ranges from 0 to 1, with higher values indicating higher levels of digital literacy. In this study, the Cronbach’s *α* coefficient of the digital literacy index is 0.878, suggesting good internal consistency among the indicators.

**Table 1:** Overview of indicators.

Variable category	Variable name	Reference layer	Variable symbol	Specific issue	Indicator name
Dependent variable	Mental health of older adults (MH)	Emotional state	MH1	Did you feel in good spirits over the past week?	Never = 1, Sometimes = 2, Often = 3
			MH2	Did you feel lonely over the past week?	Often = 1, Sometimes = 2, Never = 3
			MH3	Did you feel deeply sad over the past week?	Often = 1, Sometimes = 2, Never = 3
		Life satisfaction	MH4	Did you feel your life was going rather well over the past week?	Often = 1, Sometimes = 2, Never = 3
			MH5	Did you find much enjoyment (interesting things) in life over the past week?	Never = 1, Sometimes = 2, Often = 3
		Physical and mental condition	MH6	Did you feel you did not want to eat over the past week?	Often = 1, Sometimes = 2, Never = 3
			MH7	Did you sleep poorly over the past week?	Often = 1, Sometimes = 2, Never = 3
		Self-perception	MH8	Did you feel useless over the past week?	Often = 1, Sometimes = 2, Never = 3
			MH9	Did you feel you had nothing to do over the past week?	Often = 1, Sometimes = 2, Never = 3
Core independent variable	Digital literacy (DL)	Digital access conditions	DL1	Does your current residence have internet access (wired or wireless)?	No = 0, Yes = 1
		Basic operational skills	DL2	Do you use the internet? (Including via mobile phones and other electronic devices)	Never online = 0, Several times a year = 1, At least once a month = 2, At least once a week = 3, Daily = 4
			DL3	Does your household make payments via digital platforms such as WeChat Pay or Alipay?	No = 0, Yes = 1
		Communication and Digital Entertainment	DL4	Have you used the WeChat application (APP) in the past week?	No = 0, Yes = 1
			DL5	Have you used the Douyin application (APP) in the past week?	No = 0, Yes = 1
Control variables	Basic characteristics	Gender	SEX	What is your gender?	Female = 1, Male = 2
		Age	AGE	How old are you this year?	Age (log-transformed)
		Marital status	MAR	What is your current marital status?	1 = Unmarried, 2 = Divorced, 3 = Widowed, 4 = Married with spouse
		Care needs	CARE	Do you currently require assistance with daily living activities (such as eating, bathing, dressing, or using the toilet)?	Not required = 0, Required = 1
	Socioeconomic status	Educational level	EDU	What is your level of education?	0 = Illiterate, 6 = Primary/Literacy programme, 12 = Lower secondary, 15 = Upper secondary/Vocational, 18 = College/Higher education
		Household registration status	REG	What is your household registration status?	1 = Agricultural household registration, 2 = Non-agricultural household registration
		Annual personal income	INCOME	What was your total personal income over the past 12 months?	Yuan (log-transformed)
	Family characteristics	Number of children	CHILD	How many children do you have (including biological, adopted, fostered, and deceased)?	Number
Mediating variable	Social network (SN)	Frequency of social interaction	SN1	How many friends do you meet or contact at least once a month?	None = 0, 1 = 1, 2 = 2, 3–4 = 3, 5–8 = 4, 9 or more = 5
		Emotional support network size	SN2	How many friends can you confide in about personal matters?	None = 0, 1 = 1, 2 = 2, 3–4 = 3, 5–8 = 4, 9 or more = 5
		Instrumental support network size	SN3	How many friends can offer you help when needed?	None = 0, 1 = 1, 2 = 2, 3–4 = 3, 5–8 = 4, 9 or more = 5
Instrumental variables	Family support	Family support	FS	How many family members/relatives do you meet or contact at least once a month?	None = 0, 1 = 1, 2 = 2, 3–4 = 3, 5–8 = 4, 9 or more = 5
	Family relationships	Family relationships	FR	How many family members/relatives can you confide in about personal matters?	None = 0, 1 = 1, 2 = 2, 3–4 = 3, 5–8 = 4, 9 or more = 5

#### 3.2.3 Control variable

To control for potential confounding factors, this study selected control variables based on existing literature, covering three dimensions: individual characteristics, socioeconomic status, and family circumstances. Individual characteristics included respondents’ gender, age, marital status, and whether they required daily care from others; Socioeconomic status encompassed educational attainment, household registration status, and personal income level; physical health comprised general physical condition; while family circumstances controlled for the number of children. These variables collectively served to moderate the impact of individual heterogeneity on mental health outcomes (Huang and Ni, 2022).

#### 3.2.4 Instrumental variables

Although the baseline regression model controls for multiple dimensions of confounding factors, endogeneity may still arise in the relationship between digital literacy and mental health due to omitted variables (e.g., personality traits or cognitive ability) or reverse causality (older adults with poorer mental health may participate less in digital activities). To address these concerns, this study employs an instrumental variable (IV) approach for identification and correction. Two instruments were selected based on their relevance to the core independent variable “digital literacy” while remaining theoretically exogenous to the dependent variable “mental health”: (1) frequency of contact with family members (“How many family members or relatives do you meet or contact at least once a month?”), and (2) availability of emotional support from relatives (“How many family members or relatives can you confide in regarding private matters?”). These two variables reflect an individual’s family social capital. Frequent family interaction may facilitate older adults’ exposure to and learning of digital technologies, thereby enhancing their level of digital literacy. Meanwhile, given that the model already controls for social networks and socioeconomic characteristics, family contact is expected to influence mental health primarily through its effect on digital literacy, with its direct effect effectively constrained, thereby satisfying the exogeneity condition. The validity of the instruments will be rigorously tested in Section 4.2. Specifically, the Hansen J test will be employed to conduct an overidentification test in order to assess the exogeneity and overall validity of the instrumental variables.

Consistent with the conceptual framework, the mediating variable is operationalized as friend-based supportive social networks. This construct captures both structural (network size and interaction frequency) and functional (emotional and instrumental support exchange) dimensions of older adults’ social networks within the friends domain. Three indicators are used: (1) Instrumental support network size (“How many friends can offer you help when needed?”); (2) Emotional support network size (“How many friends can you confide in about personal matters?”); (3) Frequency of social interaction (“How many friends do you meet or contact at least once a month?”). To construct a composite index of supportive network resources, the entropy weighting method is employed. After min–max normalization, entropy values are calculated to determine indicator weights based on informational variability. The weighted aggregation of the three indicators yields the index of friend-based supportive social networks (Lin et al., 2024; Tang et al., 2022).

### 3.3 Methodology

This study aims to systematically examine the impact of digital literacy on the mental health of older adults. To ensure comprehensive and rigorous measurement, a multidimensional indicator system is constructed to capture digital literacy, while mental health is measured using the validated CES-D scale following its standard scoring procedure. In constructing the composite capability index, the entropy weighting method is employed to assign weights objectively, thereby enhancing the rationality and objectivity of the weighting scheme. The empirical analysis proceeds in several steps. First, descriptive statistics are conducted to characterize the distributional properties of the key variables and their preliminary associations. Building upon this foundation, multivariate regression models are specified, and ordinary least squares (OLS) estimation is applied to assess the effect of digital literacy on mental health outcomes among older adults. To strengthen the robustness and interpretability of the findings, additional analyses are performed. Specifically, an instrumental variable (IV) approach is implemented to address potential endogeneity concerns, and mediation analysis is conducted to explore the underlying mechanisms through which digital literacy may influence mental health. Together, these strategies enhance the credibility of the empirical identification and provide a more comprehensive understanding of the proposed relationships.

The following benchmark regression model is established in this paper (see Equation 1):


$$Y=\beta_{0}+\beta_{1}\mathrm{DL}+\beta_{2}X_{i}+\varepsilon_{1}$$ (1)

Where Y denotes the dependent variable, DL represents the core explanatory variable digital literacy, $X_{i}$ is the control variable, $\beta_{0}$ is the constant term, $\beta_{1}$ is the coefficient corresponding to the core independent variable digital literacy, $\beta_{2}$ is the coefficient for the control variable, $\varepsilon_{1}$ represents the influence of other random factors, i.e., the error term.

To further explore the mechanism through which digital literacy influences older adults’ mental health, the article introduces a mediation effect testing model, formulated as follows (see Equations 2,3):


$$M=\eta_{0}+\eta_{1}\mathrm{DL}+\eta_{i}X_{i}+\varepsilon_{2}$$ (2)

$$Y=\beta_{0}^{'}+\beta_{1}^{'}\mathrm{DL}+\mathrm{\alpha M}+\beta_{i}^{'}X_{i}+\varepsilon_{3}$$ (3)

Where M is the mediating variable, namely social network; DL is the core independent variable digital literacy; $X_{i}$ is the control variable; $\eta_{0}$ , $\beta_{0}^{'}$ are constant terms; $\eta_{1}$ , $\beta_{1}^{'}$ are the coefficients corresponding to the core independent variable digital literacy; $\eta_{i}$ , $\beta_{i}^{'}$ are the coefficients of the control variables; $\varepsilon_{2}$ , $\varepsilon_{3}$ represent the influence of other random factors, i.e., the error terms.

## 4 Results

### 4.1 Descriptive statistics

Based on analysis of 9,507 elderly respondents, the descriptive statistics for the core variables of this study are presented in Table 2. The mean composite mental health score for the elderly cohort was 20.09 points (range: 9–27 points), indicating their overall mental health status was at a moderately high level. Concurrently, the average digital literacy score was 0.203 (range: 0–1), reflecting that the current elderly population’s overall mastery of digital skills remains at a relatively low level, with considerable scope for improvement in digital participation. This baseline distribution provides crucial contextual grounding for subsequent investigations into the impact of digital literacy on mental health and its underlying mechanisms.

**Table 2:** Descriptive statistics.

Variable name	Sample size	Maximum value	Minimum value	Mean	Standard deviation
MH	9,507	27	9	20.093	2.995
DL	9,507	0.999	0	0.203	0.318
SEX	9,507	2	1	1.497	0.5
AGE (log-transformed)	9,507	4.635	4.174	4.333	0.083
EDU	9,507	16	0	6.13	3.997
MAR	9,507	4	1	3.733	0.505
REG	9,507	2	1	1.478	0.5
CARE	9,507	1	0	0.064	0.244
INCOME (log-transformed)	9,507	12.919	0	3.568	4.392
CHILD	9,507	10	0	2.418	1.317
SN	9,507	1	0	0.446	0.199

The digital literacy (DL) index was normalized using min-max scaling, resulting in a range of 0 to 1.

### 4.2 Baseline regression results

This study employed the method of least squares regression to empirically analyse the impact of digital literacy on the mental health of older adults. To ensure the appropriateness of the ordinary least squares (OLS) estimation, a series of diagnostic tests were conducted. First, variance inflation factors (VIFs) were calculated to assess potential multicollinearity among the explanatory variables. The results indicate that all VIF values are below 2, suggesting no significant multicollinearity concerns. Second, the Breusch–Pagan test was employed to examine the presence of heteroskedasticity. The test results show a chi-square statistic of 0.01 with a corresponding *p*-value of 0.9356. At the 5% significance level, the null hypothesis of homoskedasticity cannot be rejected, indicating that no significant heteroskedasticity is detected in the model. Therefore, the OLS estimates are statistically valid. Nevertheless, to further enhance the robustness of statistical inference, heteroskedasticity-robust standard errors are reported in all regression models. In addition, although the dependent variable (mental health score) is bounded within a finite range, its empirical distribution approximates normality, and the predicted values from the baseline model remain within the theoretical interval. In large samples, OLS estimators remain consistent and asymptotically unbiased even when the normality assumption of the error term is relaxed. Therefore, the use of OLS is considered appropriate and justified in the context of this study.

The regression results are presented in the table. Model 1 included only control variables, while Model 2 incorporated the core explanatory variable of digital literacy (DL) on top of this. Model 2 revealed that the coefficient for digital literacy was 0.951, and was positively significant at the 1% level. This finding indicates that enhancing digital literacy exerts a significant positive effect on the mental health of older adults. Hypothesis H1 is thus supported.

Regarding control variables, the coefficient for age was significantly negative at the 1% level (−3.971), indicating that advancing age correlates with declining mental health levels. The coefficient for educational attainment was significantly positive (0.042), suggesting that longer years of schooling correlate with higher mental health levels among older adults. The coefficient for marital status was significantly positive (0.291), demonstrating that a more favorable marital status exerts a positive influence on mental health. The coefficient for personal income was significantly positive (0.003), indicating that improved economic circumstances contribute to enhanced mental health. The coefficient for number of children was significantly positive (0.052), suggesting that older adults with more children exhibit relatively higher mental health levels. The coefficient for gender was significantly negative (−0.193), indicating that men’s mental health levels are significantly lower than women’s. The coefficient for household registration type was significantly negative (−0.332), indicating that elderly individuals with non-agricultural household registration exhibit superior mental health compared to those with agricultural registration. The coefficient for receiving care was significantly negative (−1.107), suggesting that elderly individuals capable of self-care demonstrate higher levels of mental health.

Model 2 achieved an *R*^2^ of 0.063, an improvement over Model 1’s 0.056, indicating that incorporating digital literacy enhanced the model’s explanatory power for mental health variance. In summary, digital literacy exerts a significant positive influence on elderly mental health, providing empirical evidence for understanding potential pathways to enhance mental wellbeing in the digital age. Future research may further explore specific mechanisms through which digital literacy impacts mental health, such as alleviating social isolation, strengthening social connections, or promoting active ageing (see Table 3).

**Table 3:** Least squares regression results.

Indicator		Model 1		Model 2	
Variable category	Variable symbol	Coefficient	Standard error	Coefficient	Standard error
Core independent variables	DL			0.951***	0.106
Control variables	AGE	−4.897***	0.419	−3.971***	0.432
	EDU	0.052***	0.008	0.042***	0.009
	MAR	0.305***	0.062	0.291***	0.061
	INCOME	0.065***	0.009	0.003***	0.001
	CHILD	0.135***	0.026	0.052***	0.009
	SEX	−0.182***	0.061	−0.193***	0.061
	REG	−0.285***	0.082	−0.332***	0.082
	CARE	−1.129***	0.125	−1.107***	0.125
Constant term		40.059***	1.864	36.084***	1.913
*R* ^2^		0.056		0.063	
*F*		*F*(8, 9,498) = 67.85,*p* = 0.000***		*F*(9, 9,497) = 69.11,p = 0.000***	

***, **, and * denote significance levels of 1, 5, and 10%, respectively.

### 4.3 Endogeneity tests

To mitigate potential endogeneity bias between digital literacy and mental health, this study employs an instrumental variable (IV) approach for estimation. The frequency of daily contact with relatives and the emotional support received from relatives are selected as instrumental variables for digital literacy. The Hansen J test of overidentifying restrictions yields *χ*^2^ = 3.231 (*p* > 0.05), failing to reject the null hypothesis of joint instrument exogeneity. The C statistic equals 30.17, significantly rejecting the null hypothesis that digital literacy is exogenous, indicating that the OLS estimates may suffer from endogeneity bias and that the IV approach is warranted.

After addressing endogeneity, digital literacy remains positively and significantly associated with mental health, with an estimated coefficient of 0.607. Compared with the baseline OLS estimate of 0.913, the IV coefficient is smaller, suggesting that the OLS results may be upwardly biased due to omitted variables or reverse causality. Nevertheless, after correcting for endogeneity, the core finding remains robust: digital literacy continues to exhibit a significant positive association with the mental health of older adults (see Table 4).

**Table 4:** Results of endogeneity tests.

Indicator	Coefficient	Standard error
Core independent variables (DL)	0.607***	0.137
Control variables	Controlled	
Constant term	−0.898***	0.609
*R* ^2^	0.081	
*C*-statistic	30.17***	
Hansen *J*-test	3.231	
Wald Statistic	348.298***	

*p < 0.10, **p < 0.05, ***p < 0.01.

### 4.4 Robustness tests

To further validate the robustness of the research findings, this paper altered the measurement method for the core explanatory variable, replacing the original digital literacy level calculated using the entropy method with a composite digital literacy score (DL_sum) derived from summing the indicators. Table 5 presents the regression results after variable substitution, indicating that digital literacy continues to exert a statistically significant positive influence on older adults’ mental health at the 1% level. This finding aligns with the preceding research conclusions and further substantiates the core discovery of this paper.

**Table 5:** Results of robustness test.

Indicator	Coefficient	Standard error
Core independent variables (DL_sum)	0.011***	0.001
Control variables	Controlled	
Constant term	1.467***	0.106
*R* ^2^	0.064	
*F*	*F* = 66.175, *p* = 0.000***	

*p < 0.10, **p < 0.05, ***p < 0.01.

### 4.5 Mediation effect tests

This study employs a stepwise regression approach to examine the mediating role of social networks in the relationship between digital literacy and older adults’ mental health. In addition, a bias-corrected bootstrap method (1,000 resamples) is used to test the statistical significance of the indirect effect. The results are presented in Table 5. First, Model 1 takes mental health as the dependent variable to estimate the total effect of digital literacy. The results show that digital literacy has a significantly positive effect on mental health (0.913), indicating that higher levels of digital literacy are associated with better mental health among older adults. Second, Model 2 uses social networks as the dependent variable. The results indicate that digital literacy has a significantly positive effect on social networks (0.054). Finally, Model 3 includes both digital literacy and social networks in the regression with mental health as the dependent variable. The findings show that social networks have a significantly positive effect on mental health (0.640), while the direct effect of digital literacy remains significant (0.879). Compared with the total effect (0.913), the direct effect decreases in magnitude, exhibiting a pattern consistent with partial mediation.

The indirect effect is calculated as 0.054 × 0.640 = 0.035, accounting for approximately 3.8% of the total effect (0.035/0.913). The bootstrap results show that the 95% confidence interval for the indirect effect is [0.019, 0.057], which does not include zero, indicating that the mediation effect is statistically significant. The Sobel test further supports this conclusion (*z* = 3.748, *p* < 0.01). Overall, the empirical findings are consistent with a partial mediation model, and Hypothesis H2 is supported (see Table 6).

**Table 6:** Table of mediational effect test results.

Dependent variable	Model 1		Model 2		Model 3	
	MH		SN		MH	
Indicator	Coefficient	Standard Error	Coefficient	Standard Error	Coefficient	Standard Error
Constant	36.079***	1.91	0.266**	0.129	35.909***	1.909
DL	0.913***	0.106	0.054***	0.007	0.879***	0.107
SEX	−0.188***	0.061	0.009**	0.004	−0.193***	0.061
AGE	−3.897***	0.431	0.016	0.029	−3.908***	0.431
EDU	0.042***	0.009	0.003***	0.001	0.04***	0.009
MAR	0.285***	0.061	0.025***	0.004	0.269***	0.061
REG	−0.333***	0.082	−0.012**	0.006	−0.325***	0.082
CARE	−1.028***	0.126	0.004	0.009	−1.031***	0.126
INCOME	0.050***	0.009	0.001	0.001	0.049***	0.009
CHILD	0.143***	0.026	0.006***	0.002	0.139***	0.026
SN					0.640***	0.151
*R* ^2^	0.063		0.021		0.065	
*F*	*F*(10, 9,496) = 64.953, *p* = 0.000***		*F*(10, 9,496) = 21.327, *p* = 0.000***		*F*(11, 9,495) = 60.78, *p* = 0.000***	

*p < 0.10, **p < 0.05, ***p < 0.01.

## 5 Discussion

Analysis of the sample data indicates that the overall mental health status of the elderly respondents is moderately high, whereas their proficiency in digital skills remains generally low. This pattern is consistent with Wang et al.’s (2025) assessment of digital literacy among China’s older population, highlighting a persistent structural gap in digital capability.

Regression results provide robust support for Hypothesis H1: digital literacy exerts a significant and positive influence on older adults’ mental health. This finding is aligned with existing evidence showing that digital inclusion can directly enhance both physical and psychological wellbeing among older adults (Li et al., 2025; Tang et al., 2022). In this context, digital literacy is no longer merely a technical competency; it constitutes a critical psychosocial resource that enables older adults to sustain social ties, access reliable information, and participate in digital public life. Through these channels, digital skills contribute to emotional regulation, social integration, and improved life satisfaction (Luo et al., 2025). Accordingly, at the policy level, initiatives to enhance digital literacy among older adults should be regarded as an integral component of mental health promotion rather than a narrowly utilitarian tool for improving daily convenience. Embedding digital capability-building within public health and community governance frameworks may provide a feasible intervention pathway for healthy ageing.

Further mediation analysis supports Hypothesis 2, indicating that social networks serve as a significant partial mediator between digital literacy and mental health. However, the proportion of the total effect explained by this pathway is relatively modest. One possible explanation lies in the dual structure of social support in the Chinese context, where family-based and non-family-based networks coexist. The social network measures employed in this study primarily capture peer and friend-level interactions, and may therefore not fully reflect family-based support mechanisms. As a result, the relational pathway through which digital literacy operates may represent a conservative estimate of its overall social effects. In addition, digital literacy should be understood not only as a resource for social connectivity but also as an individual psychological resource. The acquisition of digital skills may enhance self-efficacy, strengthen perceived control over information, and broaden opportunities for social participation, all of which can exert direct effects on psychological wellbeing independent of changes in network structure. Accordingly, social networks function as a partial rather than dominant mediating mechanism in the present analysis. This finding suggests that, in the context of digital transformation, the formation of mental health in later life reflects the joint influence of structural embeddedness and individual capability, and that the pathways linking digital literacy to psychological wellbeing are best understood within a more integrative theoretical framework (Zhang, 2021; Dong and Zhang, 2021).

The significant negative association between advanced age and mental health within the control variables underscores the physical and psychological challenges that tend to escalate with ageing. Socioeconomic status—measured through education and income—demonstrates a stable protective effect on mental health, reinforcing the theoretical proposition that health is shaped by long-term social determinants. Life-course socioeconomic advantages influence resource access, exposure to stressors, and opportunities for social participation, ultimately shaping wellbeing trajectories in later life. The observed protective effects of marital status and a greater number of children further indicate the enduring buffering role of traditional family support systems in China’s ageing society. Additionally, the strong association between self-care ability and mental health highlights that physical functional independence remains foundational to psychological wellbeing (Xin et al., 2025).

With respect to household registration status, agricultural hukou is significantly associated with better mental health among older adults. This finding can be interpreted in light of structural differences between urban and rural social organization in China. Compared with urban settings characterized by higher population mobility and greater functional differentiation of social ties, rural communities continue to retain relationship networks grounded in kinship and locality (Fei, 2024). The notion of the “acquaintance society” articulated by Fei Xiaotong in *From the Soil* underscores the stability and normatively embedded nature of social relations in rural China (Liu and Wang, 2018). Within such structures, individuals are embedded in dense networks of frequent interaction and reciprocal obligation, where emotional support and everyday assistance are readily accessible. This form of social embeddedness may buffer loneliness and life stress in later life, thereby fostering a more supportive psychological environment. Moreover, the marginal returns of digital technology may be particularly salient for rural older adults. When digital literacy enhances their ability to maintain long-distance connections, traditional acquaintance-based networks and newly formed digital ties may operate in a complementary manner, strengthening overall social integration.

Regarding gender differences, older men exhibit significantly lower levels of mental health than older women. This pattern can be understood not only through general gender norms but also in relation to China’s specific socio-cultural and institutional context. Within traditional family arrangements, men have typically assumed the roles of primary breadwinner and family authority. Retirement, health decline, and transformations in intergenerational family structures may weaken these role positions, potentially undermining identity continuity and perceived social value (Jessee and Carr, 2025). In contrast, women often establish and sustain kinship and neighborhood ties earlier and more consistently across the life course, resulting in social support networks that are more emotionally oriented and enduring. In later life, such relational capital may be more readily converted into psychological resilience. In addition, in the context of digital transformation, older women tend to engage more actively in affective interactions on social media platforms, which may further mitigate feelings of loneliness and social isolation (Lee et al., 2025).

## 6 Conclusion

### 6.1 Main findings

Drawing on data from the fourth wave of the China Longitudinal Ageing Study (CLASS, 2020), this study provides a systematic examination of how digital literacy shapes the mental health of older adults in China, while also identifying the broader structural factors embedded in this relationship. The findings yield several key insights.

First, digital literacy demonstrates a strong and consistent positive effect on the mental health of older adults. This result suggests that, in an increasingly digitalised society, digital competence has evolved beyond a functional skill to become an important psychosocial asset that supports emotional stability and adaptive coping in later life.

Second, the analysis shows that social networks serve as a significant partial mediator between digital literacy and mental health. Improvements in digital literacy help older adults expand interpersonal interactions and enhance their perceived social support, thereby indirectly strengthening mental health. This mechanism illustrates how digital inclusion can foster social connectedness and mitigate the risks of loneliness and isolation.

Third, the study confirms that the mental health of older adults is shaped by a constellation of socioeconomic, demographic, and family-level factors. Age, gender, educational attainment, income, marital status, number of children, and self-care ability all exhibit significant associations with mental health. Additionally, differences arising from household registration systems and gender-related socialization reveal deeper patterns of inequality in psychological adaptation and access to social support.

Overall, this study highlights the multi-layered and interdependent determinants of mental health among older adults in China. The findings contribute to a deeper understanding of how digital literacy, social networks, and structural factors jointly influence wellbeing in an ageing society. They also offer valuable empirical evidence for the design of targeted strategies to promote healthy ageing and advance Sustainable Development Goal 3, which calls for ensuring healthy lives and wellbeing for all at all ages. In the global context of accelerating digitalization and population ageing, the mechanisms identified in this study provide important theoretical and practical implications for countries—especially developing nations—seeking to build inclusive and sustainable systems that support the mental health of their older populations.

### 6.2 Limitations and future work

This study has several limitations. First, the cross-sectional design restricts rigorous causal inference and leaves open the possibility of reverse causality; moreover, the exogeneity assumption of the instrumental variable cannot be fully verified. Second, although social networks play a statistically significant mediating role, the effect size is relatively modest, suggesting that digital literacy may also influence mental health through additional psychological or cognitive pathways. Furthermore, key variables rely primarily on self-reported measures, and the social network construct does not sufficiently distinguish between family and non-family support structures. Future research should employ longitudinal or quasi-experimental designs to strengthen causal identification, incorporate multiple-mediator frameworks to expand mechanism analysis, and adopt multidimensional and more objective measurements to enhance construct validity, thereby providing a more comprehensive understanding of how digital literacy affects the mental health of older adults.

## Data Availability

The original contributions presented in the study are included in the article/supplementary material, further inquiries can be directed to the corresponding authors.
